# Supplementary material for: Stacking Model‐Based Classifiers for Dealing With Multiple Sets of Noisy Labels
Source: Biom J. 2025 Mar 12;67(2):e70042. doi: 10.1002/bimj.70042 (PMC11898607; doi:10.1002/bimj.70042)
Supplement: Supplementary file 1 — Supporting Information [file BIMJ-67-e70042-s001.zip › density-based-ensemble-model-main/competitors_real_data/netlab-master/netlab-master/help/demglm1.htm]

Netlab Reference Manual demglm1


# demglm1

## Purpose

Demonstrate simple classification using a generalized linear model.

## Synopsis

```
demglm1
```

## Description

The problem consists of a two dimensional input
matrix `data` and a vector of classifications `t`. The data is
generated from two Gaussian clusters, and a generalized linear model
with logistic output is trained using iterative reweighted least squares.
A plot of the data together with the 0.1, 0.5 and 0.9 contour lines
of the conditional probability is generated.

## See Also

`demglm2`, `glm`, `glmtrain`

---

**Pages:**
Index


---

Copyright (c) Ian T Nabney (1996-9)
